# Chemical Constituents, Antioxidant, Anti-MMPs, and Anti-Hyaluronidase Activities of *Thunbergia laurifolia* Lindl. Leaf Extracts for Skin Aging and Skin Damage Prevention

**DOI:** 10.3390/molecules25081923

**Published:** 2020-04-21

**Authors:** Wantida Chaiyana, Sunee Chansakaow, Nutjeera Intasai, Kanokwan Kiattisin, Kuan-Han Lee, Wei-Chao Lin, Shang-Chian Lue, Pimporn Leelapornpisid

**Affiliations:** 1 Department of Pharmaceutical Science, Faculty of Pharmacy, Chiang Mai University, Chiang Mai 50200, Thailand; chsunee@gmail.com (S.C.); ppp_pook@hotmail.com (K.K.); 2 Innovation Center for Holistic Health, Nutraceuticals, and Cosmeceuticals, Faculty of Pharmacy, Chiang Mai University, Chiang Mai 50200, Thailand; 3 Division of Clinical Microscopy, Department of Medical Technology, Faculty of Associated Medical Sciences, Chiang Mai University, Chiang Mai 50200, Thailand; nutjeera.in@cmu.ac.th; 4 Department of Pharmacy, Chia Nan University of Pharmacy and Science, Tainan 71710, Taiwan; lee.kuanhan@gmail.com; 5 Department of Cosmetic Science and Institute of Cosmetic Science, Chia Nan University of Pharmacy and Science, Tainan 71710, Taiwan; weilin@mail.cnu.edu.tw (W.-C.L.); myluemy@mail.cnu.edu.tw (S.-C.L.)

**Keywords:** *Thunbergia laurifolia*, matrix metalloproteinases, hyaluronidase, antioxidant, rosmarinic acid, cytotoxicity

## Abstract

This study aimed to investigate the potential usage of *Thunbergia laurifolia* Lindl. leaf extracts in the cosmetic industry. Matrix metalloproteinases (MMPs) and hyaluronidase inhibition of *T. laurifolia* leaf extracts, prepared using reflux extraction with deionized water (RE) and 80% *v/v* ethanol using Soxhlet’s apparatus (SE), were determined. Rosmarinic acid, phenolics, and flavonoids contents were determined using high-performance liquid chromatography, Folin–Ciocalteu, and aluminum chloride colorimetric assays, respectively. Antioxidant activities were determined by 1,1-diphenyl-2-picrylhydrazyl (DPPH) and linoleic acid-thiocyanate assays. MMP-1 inhibition was investigated using enzymatic and fluorescent reactions, whereas MMP-2, MMP-9, and hyaluronidase inhibition were investigated using gel electrophoresis. Cytotoxicity on human fibroblast cell line was also investigated. The results demonstrated that SE contained significantly higher content of rosmarinic acid (5.62% ± 0.01%) and flavonoids (417 ± 25 mg of quercetin/g of extract) but RE contained a significantly higher phenolics content (181 ± 1 mg of gallic acid/g of extract; *p* < 0.001). SE possessed higher lipid peroxidation inhibition but less DPPH^•^ scavenging activity than RE. Both extracts possessed comparable hyaluronidase inhibition. SE was as potent an MMP-1 inhibitor as gallic acid (half maximal inhibitory concentration values were 12.0 ± 0.3 and 8.9 ± 0.4 mg/cm^3^, respectively). SE showed significantly higher MMP-2 and MMP-9 inhibition than RE (*p* < 0.05). Therefore, SE is a promising natural anti-ageing ingredient rich in rosmarinic acid and flavonoids with antioxidant, anti-hyaluronidase, and potent MMPs inhibitory effects that could be applied in the cosmetic industry.

## 1. Introduction

The human skin is the outermost layer of the body and is the first line of defense against external pathogens and environmental insults [1]. Skin forms the most visible indicator of age [2]. Changes in skin structure occur with age-caused skin damage, e.g. wrinkle and sagging skin, which are dependent upon lifestyle and environmental exposure. Skin ageing resulting from the effects of environmental factors is known as extrinsic skin ageing, whereas skin ageing due to the passage of time is known as intrinsic skin ageing [3]. Imbalances in free radicals and proper effective action of endogenous antioxidant systems generate a condition of oxidative stress and leads to skin functional impairment, e.g., destruction of structural proteins [4]. Theses cellular changes result in signs inherent to both the intrinsic and extrinsic skin aging processes [4]. Collagen, a major extracellular matrix component in the dermis providing tensile strength, substantially changes with age [5]. Increased collagen fragmentation is thought to occur due to increasing matrix metalloproteinases (MMPs) expression in older skin [1]. Therefore, retardation and inhibition of oxidative stress and MMPs could be one of the effective mechanisms to mitigate skin ageing and skin damage. 

Since skin aging and damage are multifactorial processes, the loss of water content in the skin layer leads to a reduction of turgor, resilience, and pliability. Hyaluronic acid is the key molecule involved in skin moisture since it has a unique capacity to retain water. [6]. However, hyaluronic acid is gradually degraded by the enzyme hyaluronidase. Since hyaluronidase is the key factor that controls the turnover of hyaluronic acid in human skin [7], inhibition of hyaluronidase activity retards hyaluronic acid destruction and leads to retention of skin moisture.

*Thunbergia laurifolia* Lindl., commonly known as blue trumpet vine or laurel clock vine, is a plant in the Acanthaceae family widely distributed in Southeast Asian countries. It is believed to have detoxifying effects and has been used as a folk remedy [8]. *T. laurifolia* was reported to have antioxidant, anti-diabetic, antimicrobial, anti-inflammatory, anticancer, and antipyretic properties [8,9]. Various biologically active components were extracted from *T. laurifolia* leaves, including apigenin, caffeic acid, catechin, rosmarinic acid, rutin, isoquercetin, and quercetin [10,11]. Therefore, it was hypothesized that *T. laurifolia* may have the potential to be topically used for anti-skin-ageing in the cosmetic industry. However, the biological activities of *T. laurifolia* related to skin ageing retardation have not been reported. Consequently, we aimed to investigate the inhibitory activities of *T. laurifolia* leaf extracts against free radicals, MMPs, and hyaluronidase, which could be further applied for the prevention of skin aging and damage in cosmeceutical products.

## 2. Materials and Methods

### 2.1. Plant Materials

*T. laurifolia* leaves were obtained as dried material from Prajinburi province, Thailand. The following parameters of dried *T. laurifolia* leaves were determined: moisture content, solvent extractive value, total ash, and acid insoluble ash, following the Thai herbal Pharmacopoeia 2018 [12]. We found that the quality of the crude drug (leaves) was acceptable according to the Thai Herbal Pharmacopoeia. 

### 2.2. Chemical Materials

Collagenase from *Clostridium histolyticum* (EC.3.4.23.3), hyaluronidase from bovine testis (E.C.3.2.1.3.5), Folin–Ciocalteu reagent, rosmarinic acid, gallic acid, quercetin, α-tocopherol, (±)-6-hydroxy-2,5,7,8-tetramethylchromane-2-carboxylic acid (Trolox), 1,1-diphenyl-2-picrylhydrazyl (DPPH), ammonium thiocyanate (NH_4_SCN), sodium chloride (NaCl), calcium chloride (CaCl_2_), Alcian Blue 8GX, tricine, gelatin, hyaluronic acid, and trifluoroacetic acid were purchased from Sigma-Aldrich (Schnelldorf, Germany). Roswell Park Memorial Institute (RPMI)-1640, Dulbecco’s modified eagle medium (DMEM), L-glutamine, penicillin/streptomycin, and trypan blue were purchased from Invitrogen™ (Grand Island, NY, USA). Newborn calf serum, fetal bovine serum (FBS), and antibiotic-antimycotic 100× solution was purchased from GIBCO (Grand Island, NY, USA). Sodium dodecyl sulfate (SDS), protein markers, and Coomassie blue were purchased from Bio-Rad Laboratories (Richmond, CA, USA). Chloroform and methanol were analytical grade purchased from Labscan Asia Co., Ltd., Bangkok, Thailand. Absolute ethanol and n-hexane were analytical grade and purchased from Merck, Darmstadt, Germany. Acetonitrile was HPLC grade and purchased from Merck, Darmstadt, Germany.

### 2.3. Plant Extraction

#### 2.3.1. Continuous Solvent Extraction by Soxhlet’s Apparatus

Dried *T. laurifolia* leaves were extracted using 80% *v*/*v* ethanol with a Soxhlet’s apparatus. The resulting solvent from the Soxhlet extraction was then removed under a vacuum using a rotary evaporator (Rotavapor^®^, Büchi Labortechnik AG, Flawil, Switzerland) until dryness. An extract from Soxhlet extraction (SE) was then maintained in a refrigerator until further experiments.

#### 2.3.2. Reflux Extraction

Dried *T. laurifolia* leaves were extracted using deionized (DI) water using reflux extraction for 5 h. After the resulting solvent was left to cool to room temperature, plant residues were removed by filtering through Whatman No. 1 filter paper (Maidstone, Kent, UK). The filtrate was then concentrated by evaporation until the Brix was 3%. The remaining solvent was then removed using a Mini-Spray Dryer B-290 (Büchi Labortechnik AG, Flawil, Switzerland). An extract from reflux extraction (RE) was then maintained in a refrigerator until further experiments.

### 2.4. Rosmarinic Acid Content Determination by HPLC

Analysis of rosmarinic acid by HPLC was determined according to the method described by Junsi et al. [13]. HPLC analysis was performed on a Supelcosil LC-18 column (250 cm × 4.6 mm, 5 μm, Supelco Analytical, Bellefonte, PA, USA) as a stationary phase. The mobile phase containing (A) acetonitrile and (B) 0.1% trifluoroacetic acid in deionized water was used in gradient elution as follows: 0–50 min, 5%–80% of A; 50–60 min, 80%–80% of A; 60–61 min, 80%–5% of A; 61–100 min, 5%–5% of A, with a flow rate of 0.8 cm^3^/min at 40 °C. Each *T. laurifolia* extract was diluted in methanol and filtered through 0.45 μm nylon syringe filters (Whatman Puradisc, Healthcare Life Sciences, Buckinghamshire, UK). Twenty microliters of extract was injected into the HPLC column and was detected at 280 and 320 nm in triplicates. Various concentrations of standard rosmarinic acid solution were used to construct a standard curve for the quantitative determination of rosmarinic acid content. Subsequently, rosmarinic acid content was calculated using the following equation:

*X* = 100 (*Y* − 253,843)/34,683*Z* (R^2^ = 0.9994) (1)

where *X* is a concentration of rosmarinic acid, *Y* is an area under the curve of rosmarinic acid peak detected around 18 min, and *Z* is a concentration of *T. laurifolia* leaf extracts.

Prior to the determination of rosmarinic acid content, the HPLC method was validated for intermediate precision and percent recovery. The intermediate precision was performed by injecting three replicated injections of 100 ppm rosmarinic acid solution on two consecutive days. The relative standard deviation (RSD) was calculated to provide the analytical precision using the following equation:

*%* RSD = *SD*/*x* (2)

where % RSD is a relative standard deviation, *SD* is a standard deviation, and *x* is an average of rosmarinic acid contents triplicately analyzed on two consecutive days.

Percent recovery was performed by standard addition method after spiking of 100 ppm rosmarinic acid. Subsequently, percent recovery was calculated using the following equation:

*%* Recovery = 100*(A − B)*/*C* (3)

where *A* is a rosmarinic acid content detected after spiking of 100 ppm rosmarinic acid, *B* is a rosmarinic acid content detected before spiking of 100 ppm rosmarinic acid, and *C* is a spiking amount of rosmarinic acid. The experiments were performed in triplicate.

### 2.5. Determination of Total Phenolics Content

The total phenolic contents in the extracts were determined using the Folin−Ciocalteu assay using a calibration curve of standard gallic acid [14]. The different concentrations of the extracts were mixed with the Folin−Ciocalteu reagent and 7.5% *w*/*v* Na_2_CO_3_ was added. The mixtures were incubated for 30 min in the dark and the absorbance was measured at 765 nm using a microplate reader (SPECTROstar Nano^®^, Ortenberg, Germany). The experiments were performed in triplicate. The results are presented as gallic acid equivalent (GAE) in mg/g dry sample.

### 2.6. Determination of Total Flavonoids Content

The total flavonoid contents in the extracts were determined using the aluminum chloride colorimetric assay [14]. Quercetin served as the standard. The extracts were dissolved in ethanol at a concentration of 1 mg/cm^3^. The extract solution (25 µL) was mixed with deionized water (100 µL) and 5% sodium nitrite (10 µL). After a 5 min incubation, 10% *w*/*v* aluminum chloride and 4% *w*/*v* sodium hydroxide solution were added to the mixture. The absorbance of the solution was determined at 510 nm using a microplate reader (SPECTROstar Nano^®^, Ortenberg, Germany) and compared with the quercetin calibration curve. The experiments were performed in triplicate. Quercetin equivalent (QE) values of the extract are presented as mg quercetin/g of extract.

### 2.7. Determination of Antioxidant Activity 

#### 2.7.1. DPPH Radical Scavenging Assay

The different concentrations of extracts and standard dissolved in dimethyl sulfoxide (DMSO) were mixed with 167 μM DPPH^•^ in methanol and incubated in the dark at room temperature for 30 min [7]. Then, the mixtures were measured at 520 nm using a microplate reader (SPECTROstar Nano^®^, Ortenberg, Germany). The experiments were performed in triplicate. The results are presented as half maximal inhibitory concentration (IC_50_) values.

#### 2.7.2. Inhibition of Lipid Peroxidation Assay Using Linoleic Acid Thiocyanate Method

The method was modified from Poomanee et al. [15]. Briefly, different concentrations of all extracts and standards were dissolved in 20% *w*/*v* Tween 20. The reaction mixture consisted of linoleic acid, Tween 20, and phosphate buffer (0.2 M, pH 7.0). Then, the sample or standard was added. The peroxidation reaction was initiated by adding 46.35 mmol/m^3^ 2,2′-Azobis(2-amidinopropane) dihydrochloride (AAPH) solution in phosphate buffer into the mixture, incubated at 50.0 ± 0.1 °C in the dark for 4 h. Finally, the reaction mixtures were diluted with 75% *v*/*v* ethanol and mixed with a 20-mM FeCl_2_ solution in 3.5% HCl and 10% aqueous NH_4_SCN solution. After precisely 3 min, the degree of oxidation was determined by the absorbance measurement at 500 nm using a microplate reader (SPECTROstar Nano^®^, Ortenberg, Germany). The experiments were performed in triplicate. The results are presented as IC_50_ values.

### 2.8. Determination of Aged-Related Enzymes’ Inhibition 

#### 2.8.1. Determination of MMP-1 Inhibition by Enzymatic and Fluorescent Reactions

The different concentrations of extracts were prepared in 20% *w*/*v* Tween 20 in DI water and mixed with 0.1 mg/cm^3^ collagenase dissolved in 10 mmol/m^3^ CaCl_2_ in 125 mmol/m^3^ borate buffer, pH 7.5. The mixture was then incubated at 37 °C for 10 min. After, the collagen solution was added and incubated at 37 °C for 60 min. For the fluorescence reaction detection, the incubated solution was mixed with 0.75 mmol/m^3^ 3,4-dihydroxyphenylacetic acid (3,4-DHPAA) in DI water, 125 mmol/m^3^ sodium borate (pH 7.5), and 1.25 mmol/m^3^ of NaIO_4_ in DI water. The mixture was incubated at 37 °C for 10 min. The fluorescence intensity of the reaction mixture was measured using a SpectraMax M3 spectrofluorometer (Molecular devices, LLC, Sunnyvale, CA, USA). The excitation and emission maxima were 375 and 465 nm, respectively. The experiments were performed in triplicate. The results are presented as IC_50_ values.

#### 2.8.2. Determination of MMP-2 and MMP-9 Inhibition by Gel Electrophoresis

##### Albino Swiss Mouse Embryo Fibroblasts 3T3 Cell Culture

The 3T3 cells were seeded in T75 flasks (Corning, Oneonta, NY, USA) and then we added DMEM culture medium supplemented with 10% *v*/*v* fetal calf serum and 1% *w*/*v* antibiotic-antimycotic 100× solution. Cells were incubated in a humidified atmosphere containing 5% *v*/*v* CO_2_ at 37 °C. All culture media in the culture T75 flask were replaced with new culture media every 24 h. After the cells secreted matrix metallopeptidases-2 (MMP-2) and matrix metallopeptidases-9 (MMP-9) in the culture medium during days seven to 10, the supernatant was collected and maintained in the freezer (−20 °C) for the further investigation of MMP-2 and -9 expressions.

Various concentrations of samples were added in 96-well plates with the secretion of MMP-2 and MMP-9. The mixtures were incubated at 37 °C for 24 and 48 h. After incubation, the mixtures were collected and mixed with dye and were maintained at 4 °C for the next step.

##### MMP-2 and -9 Determination by Sodium Dodecyl Sulfate-Polyacrylamide Gel Electrophoresis (SDS-PAGE)

The levels of MMP-2 and -9 expression were investigated using SDS-PAGE [7]. The secretions of MMP-2 and -9 in the culture medium from 3T3 cell were treated with various concentrations of the extracts at 37 °C in a humidified atmosphere containing 5% *v*/*v* CO_2_. After 48 h, the treated samples were collected and mixed with an equal volume of bromophenol blue dye solution before loading in SDS-polyacrylamide gel.

For gel electrophoresis, SDS-polyacrylamide gel containing 0.1% *w*/*v* gelatin was prepared in casting chambers. The sample mixtures were then loaded into each well of SDS-polyacrylamide gel and applied directly with the voltage required to separate MMP-2 and -9. After the separation was complete, the SDS-polyacrylamide gel was removed. The gel was stained with staining buffer (Coomassie blue) and de-stained until the protein markers clearly appeared. The expressions of MMP-2 and -9 were then calculated using ImageJ 1.51J8 software (Wayne rasband, NIH, Bethesda, MD, USA).

#### 2.8.3. Determination of Hyaluronidase Inhibition by Gel Electrophoresis 

*T. laurifolia* leaf extracts were investigated for hyaluronidase inhibitory activity using gel electrophoresis [7]. The samples were mixed with hyaluronidase from bovine testis and incubated at 37 °C. The incubated mixtures were collected after 48 h and mixed with an equal volume of the bromophenol blue dye solution. The sample mixtures were then loaded into each well of SDS-polyacrylamide gel containing 0.17% *w*/*v* hyaluronic acid (HA) that was prepared in the casting chambers. The voltage was applied directly to separate the hyaluronidase. After the separation was completed, SDS-polyacrylamide gel was removed and stained with staining buffer (Alcian Blue 8GX, Sigma-Aldrich, St Louis, MO, USA). Finally, the gel was de-stained until the protein markers clearly appeared. The expression of HAase was then calculated using ImageJ (Wayne rasband, NIH, Bethesda, MD, USA). The experiments were performed in triplicate.

### 2.9. MTT (3-(4,5-dimethylthiazol-2-yl)-2,5-diphenyltetrazolium bromide) Cell Viability Assay 

The MTT cell viability assay was performed to detect the effect of *T. laurifolia* leaf extracts on BJ. BJ cells were adjusted to 4 × 10^4^ cells/cm^3^ in of 10% FBS-Eagle’s Minimum Essential Medium (EMEM) and 100 μL of cells was added into each well of a flat-bottomed 96-well plate. The cells were incubated at 37 °C under 5% *v*/*v* CO_2_ atmosphere overnight. We added 100 μL of various concentrations (0, 12.5, 25, 50, and 100 mg/cm^3^) of *T. laurifolia* leaf extracts in 10% FBS-EMEM into the well. Each concentration was performed in triplicate. We used the 10% FBS-EMEM with and without DMSO as the vehicle control and cell control, respectively. Cells were incubated at 37 °C under 5% *v*/*v* CO_2_ atmosphere for 48 h. Then, 15 μL of MTT dye solution (Sigma-Aldrich, St Louis, MO, USA) was added, and the cells were further incubated for 4 h. The supernatant was then removed, 200 μL of DMSO was added to each well, and then mixed thoroughly to dissolve the formazan crystals. The optical density was measured using an ELISA plate reader at 540 nm with reference wavelength at 630 nm. The percentage of cell survival was calculated from the values of absorbance of the test well and the control wells, using the following equation:

% Cell viability = *A*/*B* × 100, (4)

where *A* is the mean absorbance in the test well and *B* is the mean absorbance in vehicle control well. The average values of the percentages of cell survival at each concentration obtained from three independent experiments were plotted. The inhibitory concentration at 50% growth (IC_50_) of each extract was determined as the lowest concentration that could inhibit cell growth by 50% compared with the untreated culture, and the IC_20_ value of each extract was determined as the non-toxic concentration. 

### 2.10. Statistical Analysis

Statistical significance was assessed using the unpaired two-sample *t*-test and one-way analysis of variance (ANOVA) using SPSS 17.0 for Windows (SPSS Inc., Chicago, IL, USA). The level of significant difference was defined at *p* < 0.05, *p* < 0.01, and *p* < 0.001.

## 3. Results and Discussion

### 3.1. T. laurifolia Leaf Extracts

SE and RE were different in external appearance. SE was a sticky, greenish-brown semisolid mass, whereas RE was a greenish-brown dried powder. Both *T. laurifolia* leaf extracts had their own characteristic odors. The yields of SE and RE were 14.5% and 19.3%, respectively.

### 3.2. Rosmarinic Acid Content of T. laurifolia Extracts

**Figure 1:**
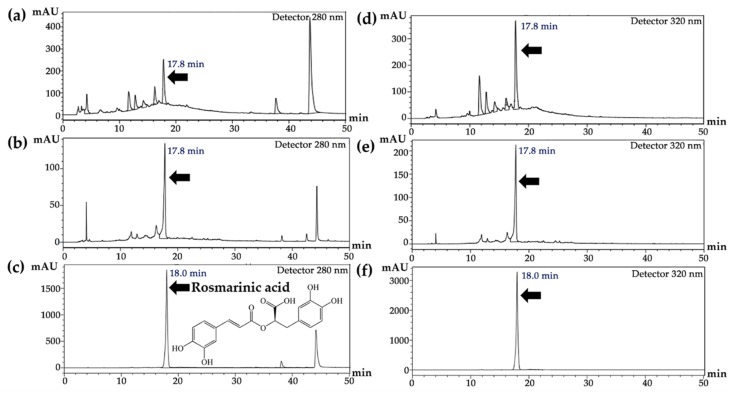
HPLC chromatograms of (**a**) *T. laurifolia* leaf extracts prepared using Soxhlet’s apparatus with 80% *v*/*v* ethanol (SE), (**b**) *T. laurifolia* leaf extracts prepared using reflux extraction with deionized water (RE), and (**c**) rosmarinic acid detected at the wavelength of 280 nm; (**d**) SE, (**e**) RE, and (**f**) rosmarinic acid detected at 320 nm.

The RSD of 0.68% and the rosmarinic acid recovery of 98–102% remarked the present HPLC method as a validated assay with good precision (RSD > 2.0%) that presented trueness data. HPLC chromatograms of SE, RE, and rosmarinic acid are shown in Figure 1. The major components of both SE and RE were defined to rosmarinic acid (5.62% + 0.01% and 1.87% + 0.02%, respectively) according to the specific retention time (18 min) of the reference standard. The results were in agreement with those reported by Suwanchaikasem et al. [16], where rosmarinic acid, a phenolic ester consisting of caffeic acid and 3,4-dihydroxyphenylacetic acid, was found to be one of the main phytochemical compounds in *T. laurifolia*. Although rosmarinic acid is a water-soluble phenolic compound [17], it dissolves better in polar organic solvents, e.g., ethanol. This is a reason why SE was more enriched in rosmarinic acid than RE.

### 3.3. Total Phenolics and Total Flavonoids Contents of T. laurifolia Leaf Extracts

Total phenolics and total flavonoids contents of *T. laurifolia* leaf extracts are shown in Table 1. We found that RE had a significantly higher phenolics content than SE (*p* < 0.01), whereas SE had a significantly higher flavonoids content than RE (*p* < 0.001). The likely explanation is the different polarities of the extracting solvents used in the extraction process, since phenolic compounds are mostly hydrophilic, but flavonoids are mostly hydrophobic [18]. Therefore, flavonoids were more easily extracted by 80% *v*/*v* ethanol than DI water. However, the results showed that the total phenolics content of *T. laurifolia* leaf extracts is not related to the content of rosmarinic acid, a phenolic carboxylic acid. The likely explanation might be the phenolic compounds other than rosmarinic acid, such as caffeic acid [19,20]. Additionally, the low specificity of the Folin–Ciocalteu method might lead to overestimated results since compounds other than polyphenols could be interfering. However, the Folin–Ciocalteu method is a simple and the most widely used spectrophotometry technique for the quantification of phenolics content [11,21,22]. 

### 3.4. Antioxidant Activity of T. laurifolia Leaf Extracts

Antioxidant activities of *T. laurifolia* leaf extracts compared to several commonly used antioxidants in the food, cosmetic, and pharmaceutical industries are shown in Table 2. Ascorbic acid (vitamin C) and α-tocopherol (vitamin E) are commonly used worldwide in various industries. Ascorbic acid was used as a representative of water-soluble antioxidants, whereas α-tocopherol was used as a representative of oil soluble antioxidants. Ascorbic acid was investigated for only DPPH^•^ radical scavenging activity and α-tocopherol was investigated for only lipid peroxidation inhibition. Trolox, a water-soluble analog of vitamin E, was investigated for both DPPH^•^ radical scavenging and lipid peroxidation inhibitory activities. Gallic acid was used as a representative of phenolic compounds and quercetin was used as a representative of flavonoids. Among various commonly used antioxidants, gallic acid was significantly the most potent DPPH^•^ radical scavenger (*p* < 0.05). In contrast, Trolox and quercetin were significantly the most potent lipid peroxidation inhibitors (*p* < 0.05).

Although *T. laurifolia* leaf extracts were not as potent as these commonly used antioxidants, they exhibited some radical scavenging and lipid peroxidation inhibition potential. RE possessed significantly higher DPPH^•^ radical scavenging activity than SE (*p* < 0.05). We observed that the DPPH^•^ radical scavenging activity of *T. laurifolia* leaf extracts related well to their phenolics content. Therefore, phenolic compounds are the major components responsible for the radical scavenging activity of *T. laurifolia* leaf extracts. These findings are well supported by the significantly highest DPPH^•^ radical scavenging activity of gallic acid. These findings support the previous reports showing that phenolic motifs highly efficiently scavenge free radicals by donating a hydrogen to the free radical and forming a stable antioxidant free radical [23]. The significantly higher DPPH^•^ radical scavenging activity of RE compared to SE was due to its greater hydrophilicity since RE was extracted using a hydrophilic solvent (DI water) compared to SE, which was extracted using a semi-polar solvent (80% *v/v* ethanol) and DPPH^•^ is more compatible with a hydrophilic system. The significantly lower lipid peroxidation inhibitory activity of RE might be due to its significantly higher phenolics content because phenolic compounds have been reported to be incompatible with the lipid peroxidation test system [24]. The results agreed well with previous studies reporting that natural extracts rich in phenolic compounds are important as natural antioxidants, e.g., apple juice, blue honeysuckle berry juice, rhubarb extracts, etc. [25,26]. In contrast, SE possessed significantly higher lipid peroxidation inhibition than RE (*p* < 0.05). The likely explanation is its higher content of flavonoids, which are hydrophobic aromatic compounds generally consisting of a flavan nucleus [27], so RE was compatible with the lipid peroxidation test system. The findings from this study support the previous reports since quercetin exhibited significantly higher lipid peroxidation inhibition than gallic acid (*p* < 0.05).

### 3.5. MMP1 Inhibition of T. laurifolia Leaf Extracts

**Figure 2:**
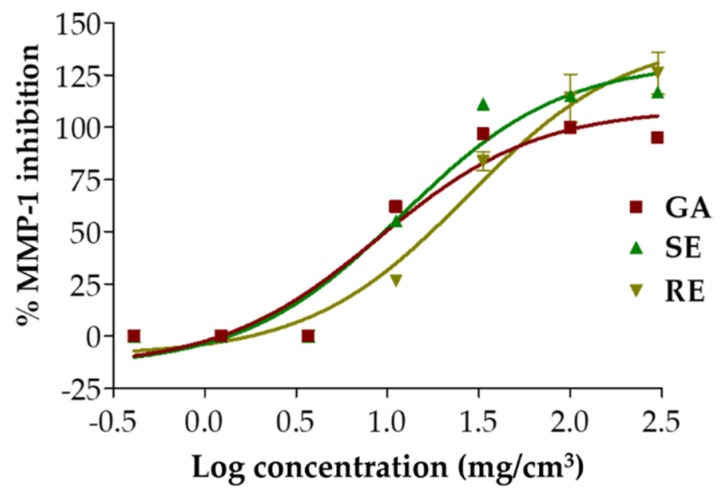
Dose response curve against matrix metallopeptidase-1 (MMP-1) of gallic acid (GA), *T. laurifolia* leaves extracted by Soxlet apparatus (SE), and *T. laurifolia* extracted by reflux extraction (RE).

**Figure 3:**
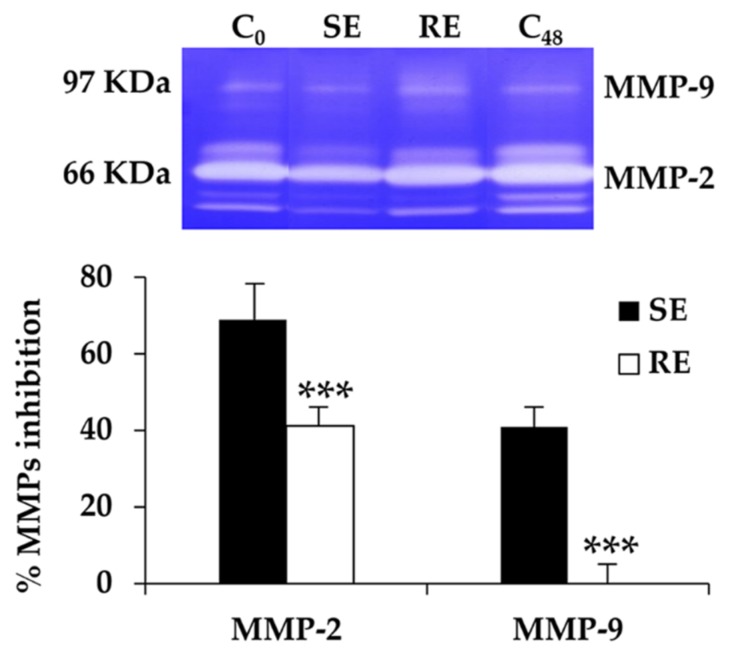
MMP-2 and -9 inhibitory activities of *T. laurifolia* leaves extracted by Soxlet apparatus (SE) and reflux extraction (RE). Asterisks denotes significant differences between SE and RE; *** *p* < 0.001.

The MMP-1 inhibitory effect of *T. laurifolia* leaf extracts is shown in Figure 2. SE possessed significantly higher MMP-1 inhibition than RE, with IC_50_ values of 12.0 ± 0.3 and 31 ± 13 mg/cm^3^, respectively (*p* < 0.05). MMP-1 inhibition of SE was as potent as gallic acid (IC_50_ = 8.9 ± 0.4 mg/cm^3^). Therefore, SE is a promising natural extract for retardation of collagen breakdown because it possesses comparable MMP-1 inhibition to gallic acid, which is a potent collagenase inhibitor [28]. Similarly, SE possessed a significantly higher MMP-2 and -9 inhibitory effect than RE, as shown in Figure 3 (*p* < 0.001). SE inhibited MMP-2 and -9 activities by 41% ± 6% and 67% ± 2%, respectively. These findings could be used to support the anti-ageing properties of SE since MMPs, in particular MMP-1, -2, and -9, can result in reduced skin elasticity and the classical signs of skin ageing [1]. Dermal fibroblasts contribute to age-associated dermal thinning as they are reduced in size due to lower pro-collagen and increased MMP-1 expressions in elderly individuals [1]. SE is suggested for further use as a natural active ingredient in cosmetic or cosmeceutical products. 

### 3.6. Hyaluronidase Inhibition of T. laurifolia Leaf Extracts

**Figure 4:**
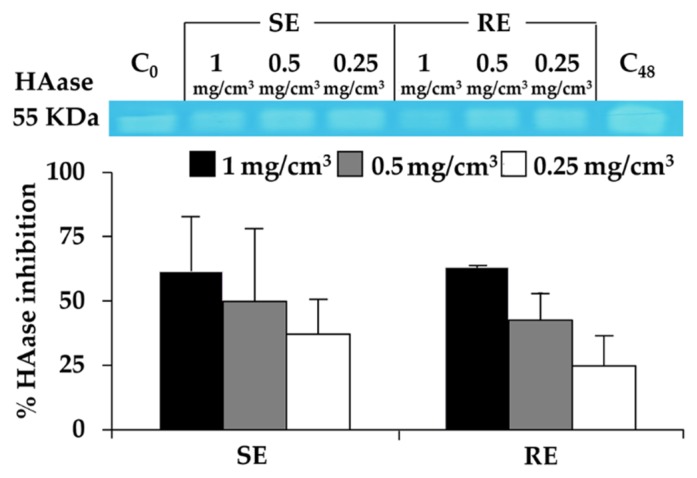
Hyaluronidase (HAase) inhibitory activities of *T. laurifolia* leaves extracted by Soxlet apparatus (SE) and reflux extraction (RE).

Since hyaluronic acid in the different skin layers has been reported to facilitate the ability to modulate skin moisture in a rational manner [6] but is easily degraded by hyaluronidase on the surface of skin [29], a hyaluronidase inhibitor would be a promising compound to remain skin moisture and prevent skin wrinkles. The hyaluronidase inhibitory activity of *T. laurifolia* leaf extracts are shown in Figure 4. Both SE and RE possessed hyaluronidase inhibitory activity in a dose-dependent manner with no significant difference (*p* > 0.05). SE inhibited hyaluronidase by 61% ± 21%, 50% ± 28%, and 3%7 ± 14% at the concentrations of 1, 0.5, and 0.25 mg/cm^3^, respectively. RE inhibited hyaluronidase by 63.0% ± 0.8%, 43% ± 11%, and 25% ± 12% at the concentrations of 1, 0.5, and 0.25 mg/cm^3^, respectively. Therefore, both SE and RE are suggested as hyaluronidase inhibitors for further development of cosmetic or cosmeceutical products.

### 3.7. Effect of T. laurifolia Leaf Extracts on Cell Viability of Human Fibroblast BJ Cell Line

**Figure 5:**
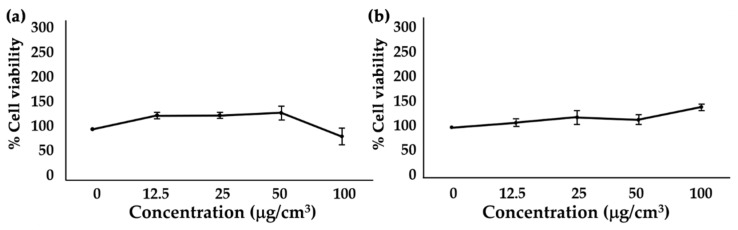
Human fibroblast BJ cell viability after treated with (**a**) SE and (**b**) RE.

Apart from biological activities, safety is another big concern. The cytotoxicities of SE and RE on human fibroblast BJ cells are shown in Figure 5. The results showed that both SE and RE did not interfere with BJ cell viability (IC_50_ > 100 mg/cm^3^). Although some previous studies reported a dose-dependent cytotoxicity of phenolic and flavonoid compounds due to their pro-oxidant properties, we found that *T. laurifolia* leaf extracts possessed antioxidant activities with no cytotoxicity effect on human fibroblast BJ cells [30,31,32]. Therefore, both SE and RE are suggested as safe natural anti-ageing components. However, further investigation on human volunteers is necessary. 

## 4. Conclusions

This is the first study to reveal the potential usage of *T. laurifolia* leaf extract in the cosmetic industry as an anti-ageing ingredient. RE yielded higher extract content (19.3%) than SE (14.5%) and also had a significantly higher phenolics content (181 ± 1 mg of GAE/g of extract) and DPPH^•+^ inhibition (*p* < 0.01). Both SE and RE inhibited hyaluronidase with comparable inhibitions of 61% ± 21% and 63.0% ± 0.8%, respectively. In contrast, SE had significantly higher rosmarinic acid content (5.62% ± 0.01%), flavonoids content (417 ± 25 mg of QE/g of extract), lipid peroxidation inhibition, and MMPs inhibition. SE possessed comparable MMP-1 inhibition to that of gallic acid with IC_50_ values of 12.0 ± 0.3 and 8.9 ± 0.4 mg/cm^3^, respectively (*p* > 0.05). Therefore, SE is suggested as natural anti-ageing ingredient with antioxidant, anti-hyaluronidase, and potent inhibitory activities against MMPs.

## Figures and Tables

**Table 1:** Total phenolics and total flavonoids content of *Thunbergia laurifolia* leaf extracts.

Sample	Total Phenolic Content (mg of Gallic Acid/g of Extract)	Total Flavonoids Content (mg of Quercetin/g of Extract)
SE ^1^	174 ± 2	417 ± 25 ***
RE ^2^	181 ± 1 **	270 ± 10

^1^ SE, *T. laurifolia* extracted by Soxlet apparatus; ^2^ RE, *T. laurifolia* extracted by reflux. Asterisks denotes significantly different between SE and RE; * *p* < 0.01, *** *p* < 0.001.

**Table 2:** Antioxidant activity of *T. laurifolia* leaf extracts.

Sample	Half Maximal Inhibitory Concentration (IC_50_: μg/cm^3^)	
	1,1-diphenyl-2-picrylhydrazyl (DPPH Inhibition)	Lipid Peroxidation Inhibition
Ascorbic acid	4.4 ± 0.3 ^b^	N.D.
α-Tocopherol	N.D.	4.3 ± 0.3 ^c^
Trolox	6.8 ± 0.6 ^c^	0.2 ± 0.0 ^a^
Gallic acid	1.8 ± 0.0 ^a^	1.2 ± 0.1 ^b^
Quercetin	2.7 ± 0.5 ^a,b^	0.1 ± 0.0 ^a^
SE	217 ± 8 ^e^	8 ± 1 ^d^
RE	89 ± 1 ^d^	12.9 ± 0.1 ^e^

N.D., not determined. a, b, c, d, and e denote significantly different among samples at *p* < 0.05.
